# Exploration of microbial diversity in the ethnic starter culture, Balam through 16S and ITS amplicon sequencing

**DOI:** 10.1128/mra.01394-25

**Published:** 2026-10-08

**Authors:** Shikha Tomar, Kumud Pant, Promila Sharma, Sujay Ayyagari, Rajsekhar Adhikary

**Affiliations:** 1 Department of Microbiology, Graphic Era Deemed to be University 209257, Dehradun, Uttarakhand, India; 2 Department of Biotechnology, Graphic Era Deemed to be University 209257, Dehradun, Uttarakhand, India; 3 Luddy School of Informatics and Computing, IUPUI 10668 , Indianapolis, Indiana, USA; 4 Department of Botany, Murshidabad University 338914, Berhampore, West Bengal, India; Fluxus Inc., Sunnyvale, California, USA

**Keywords:** 16S and ITS amplicon sequencing, Balam, microbial population, starter culture, traditional fermentation

## Abstract

Amplicon sequencing of 16S rRNA and ITS of the traditional amylolytic starter culture Balam from Uttarakhand revealed a diverse microbial community dominated by the bacterial genera *Lactobacillus* and *Pseudomonas* and the fungal genus *Saccharomycopsis*. These fermentative microorganisms likely enhance indigenous fermentation efficiency and product palatability.

## ANNOUNCEMENT

Balam, a traditional back-slopped starter culture from Uttarakhand, India, is used for fermenting beverages such as Jaan and Kacchi (1). Prepared from roasted wheat flour and spices, its fermentation is driven by yeasts and lactic acid bacteria, although culture-dependent studies have identified only limited taxa, overlooking unculturable microbial diversity (2–4). Therefore, this study employed 16S rRNA and Internal Transcribed Spacer (ITS)-based Illumina amplicon sequencing to comprehensively characterize the microbial diversity of Balam from Johar Valley, District Pithoragarh, Uttarakhand, India (29°34′58.2996″ N, 80°13'5.4660″ E).

Metagenomic DNA was extracted from 200 mg of Balam starter culture using the QIAamp PowerFecal Pro DNA Kit (Qiagen, Germany; Cat. No. 51804) according to the manufacturer’s instructions. DNA quality was assessed by agarose gel electrophoresis, while concentration and purity were determined using a NanoDrop spectrophotometer. Samples with an A260/280 ratio of 1.8–2.0 were selected for downstream analysis. The bacterial 16S rRNA gene was amplified using universal bacterial 16S rRNA gene primers (forward primer 5′-AGAGTTGATGMTGGCTCAG-3′ and reverse primer 5′-TTACCGCGGCMGCSGGCAC-3′) and ITS primers (forward primer 5′-TTGGTCATTTAGAGGAAGTAA-3′ and reverse primer 5′-GCTGCGTTCTTCATCGATGC-3′). PCR comprised an initial denaturation at 95°C for 3 min, followed by 25 cycles of 95°C for 15 s, 60°C for 15 s, and 72°C for 2 min, with a final extension at 72°C for 10 min. Amplicons were purified using AMPure XP beads, indexed with Illumina barcoded adapters (8-cycle PCR), quantified using the Qubit dsDNA HS assay, and sequenced on the Illumina MiSeq platform (2 × 300 bp). Raw sequence quality was assessed using FastQC v0.11.2 and MultiQC v1.9. Reads were trimmed with Trim Galore, followed by paired-end merging, chimera removal, OTU clustering at 97% similarity, and taxonomic assignment against the SILVA 138 database using QIIME2 v2022.2 (5, 6). All bioinformatic analyses were performed using default parameters.

**Fig 1:**
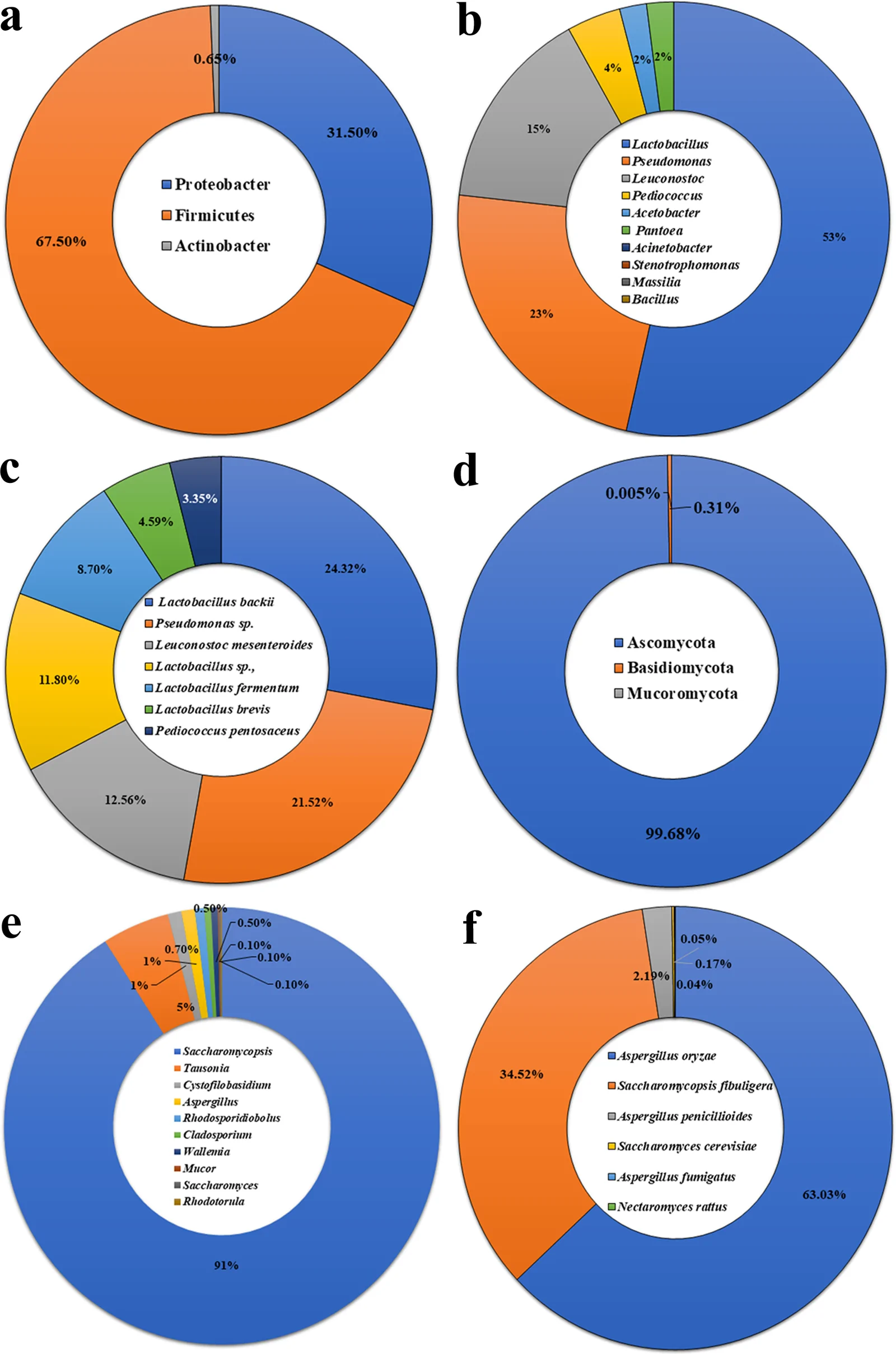
Relative abundance of bacterial diversity on the (**a**) phylum, (**b**) genus, and (**c**) species levels and fungal diversity (**d**) on the phylum, (**e**) genus, and (**f**) species levels.

Phred score confidence established high-quality reads of 16S rRNA and ITS data sets, with amplicon sequencing resulting in approximately 116.5 and 45.9 million bases, respectively, and high-quality reads having Phred quality scores above Q30. Firmicutes (67.50%) dominated at the phylum level, with the next largest proportion of 31.50 going to Proteobacteria and 0.65 to Actinobacteria. Analysis at the genus level showed that the predominant organisms were *Lactobacillus* (53%), *Pseudomonas* (23%), *Leuconostoc* (15%), and *Pediococcus* (4%). On the species level, *Lactobacillus backii* (24.32%), *Pseudomonas* sp. (21.52%), and *Leuconostoc mesenteroides* (12.56%) were the most commonly found (Fig. 1), reflecting roles in fermentation, texture formation, probiotic activity, and in industrial applications (7–9). The fungal microbiome contained also a total of 34.5% guanine–cytosine (GC) content dominated by Ascomycota (99.68%). Key genera *Saccharomycopsis* (91%) and *Tausonia* (5%). *Aspergillus oryzae* (63.03%) and *Saccharomycopsis fibuligera* (34.52%) were the most common species that are industrially relevant (Fig. 1), along with the enzyme-producing *A. oryzae* and starch-converting and single-cell protein (SCP)-producing *S. fibuligera* (10, 11). In general, this metagenomics study of Balam indicated a variety of culturable and unculturable taxa, such as probiotic LAB and functional yeasts, which are synergistically driving carbohydrate degradation, acidification, and alcoholic fermentation traditionally.

## Data Availability

The bacterial 16S rRNA sequencing data are available in the NCBI Sequence Read Archive (SRA) under accession number SRR27876490, while the fungal ITS amplicon sequencing data are available under accession number SRR38517601. Both data sets are associated with BioProject accession PRJNA1071288 and BioSample accession SAMN39683560.
